# Using Neuroscience to Inform Tobacco Control Policy

**DOI:** 10.1093/ntr/nty057

**Published:** 2018-03-24

**Authors:** Olivia M Maynard, F Joseph McClernon, Jason A Oliver, Marcus R Munafò

**Affiliations:** 1 UK Centre for Tobacco and Alcohol Studies, School of Experimental Psychology, University of Bristol, Bristol, United Kingdom; 2 MRC Integrative Epidemiology Unit (IEU), School of Experimental Psychology, University of Bristol, Bristol, United Kingdom; 3 Department of Psychiatry and Behavioral Sciences, Duke University Medical Center, Durham, NC

## Abstract

**Introduction:**

Techniques employed in the field of neuroscience, such as eye tracking, electroencephalography, and functional magnetic resonance imaging, have been important in informing our understanding of the cognitive mechanisms underlying tobacco smoking. These techniques are now increasingly being used to investigate the likely impact of tobacco control policies.

**Aims and Methods:**

In this narrative review, we outline the value of these methodological approaches in answering policy-relevant tobacco control research questions, with a particular focus on their use in examining the impact of standardized cigarette packaging and health warnings. We also examine the limitations of these methodologies and provide examples of how they can be used to answer other policy-relevant questions.

**Results:**

We argue that neuroscience techniques can provide more objective evidence of the impacts of policy measures, allow investigation where it is not possible to conduct behavioral manipulations, and facilitate a deeper understanding of the cognitive mechanisms underlying the impacts of tobacco control policies such as standardized packaging, health warnings, point-of-sale displays, and mass media campaigns.

**Conclusions:**

Rather than replacing more traditional methods of examining tobacco control measures, such as observational experiments, surveys, and questionnaires, neuroscience techniques can complement and extend these methods.

**Implications:**

Neuroscience techniques facilitate objective examination of the mechanisms underlying the impacts of tobacco control measures. These techniques can therefore complement and extend other methodologies typically used in this field, such as observational experiments, surveys, and questionnaires.

Techniques commonly employed in the field of cognitive neuroscience (described here as neuroscience techniques) are increasingly being used to investigate the likely impact of tobacco control measures. The overarching premise of our narrative review is that these techniques can complement and extend self-report and behavioral research approaches commonly used to evaluate tobacco control measures. First, we describe how neuroscience techniques can overcome some of the limitations of these other research techniques. Second, we provide a review of studies using eye tracking, electroencephalography (EEG), and functional magnetic resonance imaging (fMRI; see Box 1 for a more detailed description of these techniques) to evaluate these policies, with a particular focus on standardized packaging and health warnings. Third, we discuss the limitations of these techniques. We conclude with a discussion of the future of neuroscience techniques for tobacco control research and provide a framework for future research.

## Overcoming the Limitations of Other Methodologies

Every research methodology has its limitations (and we discuss limitations with neuroscience techniques later). However, techniques used in neuroscience can overcome some of the limitations associated with methodologies often used to examine questions related to tobacco control policies, including those relying on subjective responses.

### Providing More Objective Measures

Traditionally, research examining the potential effectiveness of tobacco control measures has relied on subjective response methodologies, such as surveys, focus groups, and questionnaires. A recent systematic review of 68 experimental studies comparing text-only warnings with pictorial health warnings identified a total of 278 outcome measures characterizing 61 constructs across these studies.^1^ Of these, only 23 outcomes were objective (visual attention *n* = 10, recall/recognition *n =* 5, response time *n =* 3, quitline calls *n* = 1, and smoking behavior *n* = 4). Despite considerable converging evidence,^2,3^ tobacco industry advocates argue that approaches relying on subjective reports do not constitute “credible evidence.”^4^ Tobacco industry criticisms on the grounds of a lack of credible evidence are likely to have slowed United Kingdom government decision-making on standardized packaging^5,6^ and US decision-making on health warnings.^7^ Indeed, as intentions are known to only play a small role in actual behavior,^8^ research which relies on self-reported outcomes has also been criticized by academics within the field of tobacco control who have argued that experimental designs with reliable, behavioral measures are required to provide “convincing evidence.”^9^ Neuroscience techniques allow us to examine processes and behaviors that are outside of the awareness of individuals and therefore are not subject to these same criticisms.

### Facilitating Investigation Where Behavioral Manipulations Are Not Possible

Behavioral research methodologies can indeed provide more convincing evidence of the effects of a proposed tobacco control measure. A recent randomized controlled trial evaluated the effects of pictorial cigarette pack warnings on quit attempts,^10^ while another has examined the impact of standardized packaging on actual smoking behavior.^11^ Similarly, in a recently completed randomized controlled trial, the effects of reducing the nicotine content in cigarettes on smoking behavior over 6 weeks were evaluated in a diverse sample of US smokers.^12^ The results provide more compelling evidence than would a survey study asking smokers to self-report on their opinions or imagined reactions to a reduced-nicotine cigarette. However, while studies with behavioral outcomes provide a more rigorous evaluation of potential policy measures than those relying on subjective reports, these evaluations can take years to complete and require large sample sizes and long follow-up periods in order to observe the downstream effects of these interventions.^13^

In addition, behavioral outcomes are not possible when the policy manipulation is outside the control of the experimenter. For example, it is typically not feasible to randomly assign half of a country to a tobacco control measure such as standardized packaging and compare its effects on smoking outcomes to the half of the population continuing with normal practice. Instead, policymakers will wait for evidence from other countries that have introduced a tobacco control measure before implementing it themselves. For example, the United Kingdom, Ireland, and France waited for pre/postevaluations of the effectiveness of standardized packaging in Australia^14–16^ before implementing it. However, this is not always feasible, and generalizing from one country to another is an important limitation of this kind of evaluation. Neuroscience techniques can provide objective evidence of the likely effects of a tobacco control measure but in a considerably shorter timescale.

### Allowing a Deeper Analysis of Cognitive Mechanism

Studies with behavioral outcomes, while providing evidence regarding the *impact* of policy on target endpoints, are also limited in their ability to explain *why* a policy intervention worked in the manner it did. Neuroscience techniques have been important in informing our understanding of the cognitive mechanisms underlying tobacco smoking, including nicotine withdrawal,^17,18^ the impact of quitting smoking,^19^ prediction of cessation outcomes,^20–23^ and cue reactivity.^24–26^ These techniques are increasingly being used to examine the cognitive mechanisms underlying responses to tobacco control measures. In some cases, insights into cognitive mechanism may be obvious (eg, nicotine is the primary psychoactive component of tobacco smoke, and reducing it to negligible levels decreases smoking reinforcement) or unimportant. However, in other cases, a deeper understanding of how a policy manipulation exerts its influence can result in insights necessary for further refinement and even greater impact. For example, understanding *why* a particular health warning is effective at encouraging thoughts about quitting can inform development of future health warnings. Furthermore, while a group of participants in a study may all exhibit the same self-reported or behavioral response to one stimulus or manipulation, the underlying cognitive and neural processes by which they arrive at those outcomes may vary in meaningful ways.

## The Use of Neuroscience Techniques in Tobacco Control Research

By measuring processes and behaviors that are outside of the awareness of individuals, neuroscience techniques can overcome many of the limitations of methodologies relying on both subjective and behavioral responses. These techniques include eye tracking, EEG, and fMRI and are commonly used in market research to understand how neuroscience can inform consumer decision-making and *increase* purchasing behavior.^27–29^ Box 1 describes these techniques in more detail, and in the following sections, we provide a review of studies using these techniques to answer tobacco policy-relevant questions, with the goal of ultimately *reducing* purchasing of tobacco by reducing smoking initiation and encouraging cessation.

Box 1. Neuroscience Techniques Explained **Eye tracking**

Attention to a stimulus is recognized as an important prerequisite for behavior change.^54^ While early eye trackers were invasive and relied on direct and invasive observations of the eye,^80^ today’s eye trackers typically measure eye movements using noninvasive optical tracking with a video-based eye tracker. At the most basic level, eye movements can be divided into fixations (when the eye is stable in a particular location) and saccades (when it is moving to another location). Researchers typically examine the location of the first fixation, the duration of fixations, or the number of fixations. These measures provide an indication of visual attention toward a stimulus (ie, a tobacco health warning). Eye trackers are either “static” or “mobile.” When using a static eye tracker, the participant is *static* and views 2D stimuli on screen (either still or moving images). By contrast, using a mobile eye tracker allows for analysis of eye movements when individuals are *mobile* in naturalistic or real-world environments.

Eye tracking can be used to answer questions such as “Which elements of pro/anti-tobacco messages capture attention?” “How is attention allocated in tobacco retail environments?” “How does attention to vaping cues influence later smoking behavior?”

**Electroencephalography**

During EEG, electrodes are placed over the skull to measure changes in the electric field being produced by the brain. Event-related potentials (ERPs), which reflect the specific electrical activity related to a specific sensory “event” (ie, seeing an image on a computer screen), can be measured using EEG. ERPs can refer to different components, such as P300, where each component is typically quantified by its amplitude and latency related to the onset of the sensory event. Although EEG has poor spatial resolution (ie, it is difficult to determine exactly where in the brain the electrical activity is coming from), EEG affords excellent temporal resolution (within the millisecond range), meaning that it is useful for measuring brain activity over time. A large volume of literature has previously used EEG to explore the mechanisms underlying tobacco addiction and the P300 ERP, which is related to attentional processes, and participant arousal state^81^ has been reliably shown to reflect the heightened incentive value of smoking cues among smokers.^82^

EEG can be used to answer questions such as “Do anti-tobacco advertisements successfully elicit emotional responses?” “What are the neural indices underlying smokers’ reactance to health warnings?” “What is the minimum nicotine content needed to induce change in neural activity?”

**Functional magnetic resonance imaging**

The basic principle behind functional magnetic resonance imaging (fMRI) is that when a region of the brain is more active, it uses more oxygen, resulting in an increased blood flow to this region. fMRI works by detecting these changes in blood oxygenation and flow and measures blood oxygenation–level dependent (BOLD) signal in the brain. Unlike EEG, fMRI has relatively poor temporal resolution (~1 s) because of the slower speed of blood when compared with electrical impulses. However, the spatial resolution of fMRI (approximately 1–2 mm) is much greater than EEG, meaning that fMRI can be used to create maps that show the activated regions of the brain during certain tasks. fMRI is therefore important in understanding the neural mechanisms underlying behavior change.

fMRI can be used to answer questions such as “How do tobacco advertisements influence neural responses to reward?” “What effect does vaping during scanning have on neural responses related to reward and craving?” “How do neural responses to anti-tobacco messages predict later choice of tobacco products above and beyond self-report?”

### Eye Tracking

Eye tracking has been used for decades by consumer marketers, from studies examining visual attention to adverts in the Yellow Pages^30^ to those exploring the cognitive mechanisms underlying supermarket choices.^31^ This methodology provides an objective measure of what visual stimuli or objects individuals are attending to, and which aspects of the environment capture attention. The value of this research technique is also recognized by the tobacco industry, who have used mobile eye tracking (see Box 1) to examine the impact of tobacco point-of-sale displays on visual attention.^32^ Similar research has been conducted by academics.^33^

One of the mechanisms through which standardized packaging is expected to be effective is by increasing the salience of health warnings.^2,3^ Research using eye-tracking technology has therefore been particularly valuable in objectively measuring visual attention to health warnings on branded and standardized packaging,^34–38^ observing that standardized packaging increases visual attention to warnings among adult and adolescent nonsmokers and nondaily smokers.^34,35,37^ Other research has found that this is observed for text-only warnings and pictorial health warnings on standardized packs.^38^ Eye-tracking research was cited in the legal case between British American Tobacco and the UK Department of Health regarding the legality of standardized packaging legislation^39^ and was described as using “objective physiological techniques.” This research adds weight to and extends findings obtained using other research methodologies, including focus groups,^40,41^ quantitative surveys,^42,43^ and naturalistic studies,^44,45^ which have reported similar effects.

**Figure 1.**
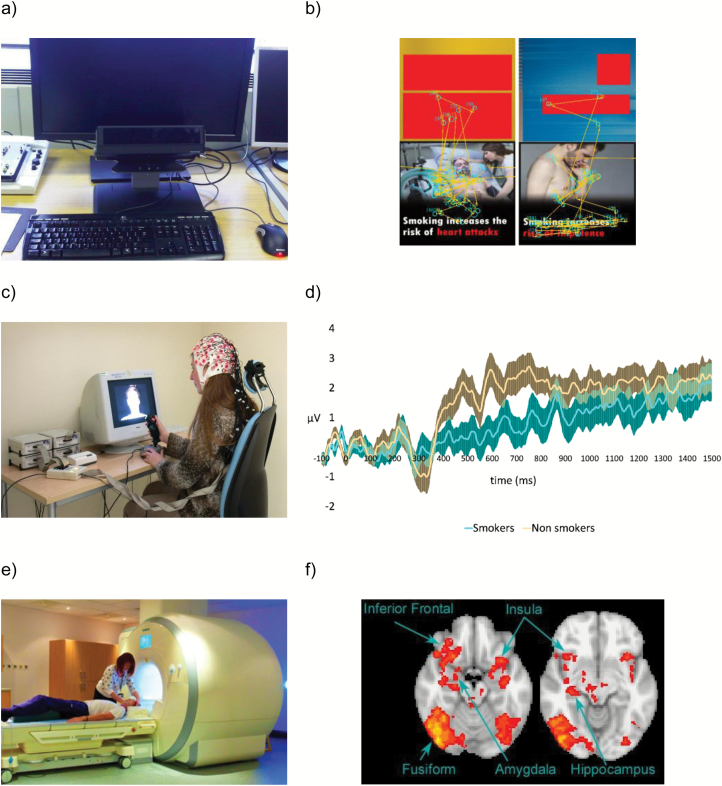
(a) Typical desk-mounted eye tracker setup (image from Flickr—https://flic.kr/p/7YWwiE); (b) eye-tracking data from a single participant showing the focus of visual attention when viewing two different cigarette packs (cigarette packs photographed by OMM and branding information blanked out). Unpublished data taken with permission from OMM; (c) typical EEG setup (image with permission from the School of Experimental Psychology, University of Bristol); (d) difference waveforms (ie, responses to health warnings subtracted from responses to control stimuli) illustrating greater emotional response (as measured using the late positive potential) to warnings for nonsmokers versus daily smokers. Data taken with permission from Stothart et al.^55^; (e) typical fMRI setup (image with permission from the School of Experimental Psychology, University of Bristol); and (f) brain activation associated with processing of high emotional reaction (ER) compared with low ER warnings. Data taken with permission from Wang et al.^62^

By measuring which components of a health warning smokers are attending to and consequently which are impacting their self-reported reactions, eye-tracking technology allows greater understanding of the cognitive mechanisms underlying the influence of tobacco marketing materials on self-reported reactions and behavioral effects (see Figure 1a and b and Box 1 that demonstrate how eye tracking can be used to examine attention to discrete regions of a cigarette pack advertisement). This is particularly important, as eye-tracking research suggests that daily smokers avoid currently used warnings,^36^ indicating that different strategies for capturing attention are required among this population. Studies using this technique have examined the impact of varying a number of different health warning components including novelty,^46,47^ size,^48^ format,^49,50^ and emotional content^51^ on visual attention. Other research has used eye tracking to examine the impact of risk statements,^52^ message congruency,^53^ and pictorial warnings^49^ in cigarette advertisements on visual attention.

### Electroencephalography

Despite considerable research showing that pictorial warnings are more effective than text-only warnings,^10,54^ the literature on which *types* of pictorial warnings are more effective and the psychological processes underlying responses to them is less well understood. For example, a number of self-report studies have found that pictorial warnings evoke both positive (ie, increased warning credibility and cognitive elaboration) and negative responses (ie, negative affective reactions and psychological reactance).^54^ By examining correlations between self-reported reactions and brain activation, EEG and specifically examination of particular event-related potentials (see Box 1) have allowed us to begin to understand the cognitive mechanisms underlying these self-reported responses.

Stothart et al.^55^ used EEG to understand the cognitive mechanism underlying avoidance of health warnings among smokers, a finding previously observed using both self-report^56^ and eye-tracking^36^ research. While smokers showed no differences in early perceptual processing when compared with nonsmokers (as indexed by the P1, P300, and visual mismatch negativity event-related potentials), they showed reduced later cognitive responses to the warnings (as indexed by reduced late positive potential, an event-related potential which is modulated by stimulus emotional intensity) when compared with nonsmokers. This suggests that smokers’ avoidance of health warnings is not due to differences in perceptual processing of the health warnings, but rather due to reduced sensitivity to the emotional content of health warnings (see Figure 1d).

In a similar EEG study, Wang et al.^57^ found that presentation of health warnings rated high on a scale of emotional reaction prior to the presentation of a smoking-related cue reduced the subsequent P300 amplitude when compared with those warnings rated lower on the emotional reaction scale (see Box 1 for a description of the P300 event-related potential). Similarly, a recent study by Cochran et al. observed that disgust-based health warnings, but not health anxiety-based warnings, reduced attentional processing of smoking cues (as indexed using EEG).^58^ Together, these studies indicate that although smokers may avoid health warnings, graphic warnings that evoke a strong emotional reaction (such as those proposed for use in the United States) may be effective at changing relevant behaviors (ie, cue reactivity), thus providing new insights into the cognitive mechanisms underlying the effects of health warnings.

### Functional Magnetic Resonance Imaging

Two previous studies have used fMRI to examine the impact of standardized cigarette packaging on neural activation. While one study observed no differences in neural activation for branded when compared with standardized packs among daily smokers,^59^ another which combined fMRI with eye tracking observed that when taking visual attention to health warnings into account, standardized when compared with branded packaging increased activation in the visual cortex, suggesting that standardized packaging increases the visual salience of health warnings.^60^ fMRI has also been used to examine neural activity when smokers are presented with cigarette package health warnings and have found that pictorial warnings activate large-scale neural networks including the hippocampus, fusiform gyrus, and supplementary motor area^61^ and the amygdala, medial prefrontal cortex, medial temporal lobe, and occipital cortex^59^ (see Figure 1f). In another fMRI study, warnings rated as being of higher emotional salience resulted in increased activation in the amygdala, hippocampus, and inferior frontal gyrus when compared with those rated as being of lower emotional salience.^62^ Together, these fMRI studies support research using qualitative and observational techniques and demonstrate the potential impact of standardized packaging and tobacco health warnings on behavior and the processes underlying these behavioral effects.

Future research in this field can draw on well-validated neural indices (ie, those related to valuation of products) to provide an objective assessment of the effects of product features such as health warnings on behavior (ie, the actual value assigned to that product). The ventromedial prefrontal cortex, for instance, has been shown to index the valuation of products across multiple different attributes of goods.^63^ In a study by Knutson et al.,^64^ brain activation in insula, ventral striatum, and medial prefrontal cortex during the evaluation of a product predicted later choice to purchase the product above and beyond self-reported preference for the product. Similar studies, but with tobacco products, could evaluate the influence of product attributes (eg, warning labels, standardized packaging) on product valuation, as well as allow for the comparison of valuation across product categories (eg, e-cigarettes vs. conventional cigarettes). The potential of this mixed methodological approach in the field of tobacco control has been demonstrated by Falk et al. who found that the extent to which antismoking messages activated the medial prefrontal cortex predicted the success of those same antismoking messages at the population level.^65^ Combining neural data with self-reported responses to the campaign accounted for the largest proportion of the variation in the population-level success of the campaign, demonstrating the added value of the neural predictor.^65^ Similarly, activation in the medial prefrontal cortex has been shown to be predictive of successful smoking cessation (as measured using expired carbon monoxide) over and above self-reported intentions to quit, self-efficacy, and the ability to relate to health messages.^66^ This technique could therefore be used to augment self-report measures which are typically used as predictors of behavior and may be particularly useful when self-reported preferences and actual behavior are likely to diverge (ie, due to social desirability bias).

## Limitations of Neuroscience Techniques and Possibilities for the Future

Although these neuroscience research techniques can be valuable adjuncts to other experimental and qualitative research and can provide important insights into the potential cognitive mechanisms underlying the effectiveness of tobacco control measures, they also have some important limitations.

### Ecological Validity

While these techniques afford excellent internal validity, allowing researchers to manipulate different elements of the tobacco control measure (ie, the type of health warning or the color of the “standardized” packaging), their ecological validity is limited. However, these limitations are not insurmountable. Indeed, there is a long history of using these techniques in market research to answer questions related to consumer behavior.^27–29^

Purchasing behavior can be modeled during fMRI by giving subjects a set amount of money that can be allocated across purchasing decisions with later, real-world delivery of those products.^64^ Recently, researchers have conducted the first feasibility trial demonstrating the use of an e-cigarette during fMRI scanning.^67^ Furthermore, with improvements in technology, neuroscience techniques are increasingly being taken into real-world environments. For example, mobile EEG^68^ and eye tracking^33,69^ equipment allow us the possibility of examining neural responses and visual attention in the real world, such that the impact of real, 3D cigarette packs on visual attention to health warnings can be assessed. In addition, a growing number of studies are bringing real-world, personally relevant stimuli into the lab and scanning environment for assessment of subjective and neural reactivity^70,71^; in the future, such methods could be applied to the study of real-world tobacco retail and point-of-sale environments. Furthermore, recent advances in virtual reality (VR) technology mean that some of the limitations of these neuroscience techniques can be overcome even in controlled laboratory settings. Using VR headsets, participants can be immersed in life-like scenes in combination with neuroscience techniques. This technology has been used to investigate the impact of smoking cues in VR environments on craving,^72^ and as a result, the impact of VR smoking cue exposure therapy on smoking cessation success.^73^ Moreover, at least three studies have evaluated craving and tobacco product purchasing behaviors in virtual tobacco retail outlets^74–76^ As far as we are aware, however, VR has yet to be combined with simultaneous eye tracking, EEG, or fMRI, or other neuroscience techniques, to answer questions relevant to tobacco control.

### Generalizability

In addition to relatively poor ecological validity, the degree to which findings from neuroscience research are generalizable is also a potential limitation. Indeed, it is important to determine to what extent the behaviors observed using neuroscience techniques are predictive of *actual* behavioral responses to tobacco control measures. For example, the extent to which visual attention to health warnings^34–38^ or neural responses to standardized packaging^59^ replicate that observed when a smoker pulls a cigarette pack from their pocket is unknown. Studies that use a combination of methodological approaches can attempt to bridge this gap. Neuroscience measures should be used in combination with questionnaires or biological measures (such as cotinine) to understand the relationship between the neuroscience measure and actual behavior. As described in the fMRI subsection, neural measures are increasingly being used to improve predictions of decision-making models, where neural activation predecision can predict later choice.^64^

### Other Considerations

Finally, each of the neuroscience techniques described here has several inherent limitations that researchers interested in their use should consider. These include high set-up costs and large amounts of time and skill required to run experiments and analyze the data. This technical obstacle can be overcome by forming collaborations between researchers with different research backgrounds. For a more detailed description of the techniques that have been described here, including their advantages and disadvantages, readers should refer to the review by Kable,^77^ which also outlines other techniques that have not been mentioned here, including magnetoencephalography, positron emission tomography, near-infrared spectroscopy, transcranial magnetic stimulation, and transcranial direct current stimulation.

## Conclusions

This review has focused on the role of neuroscience techniques in addressing research questions related to tobacco control policies, in particular, standardized packaging and health warnings. Other possibilities for future research are also discussed in Box 1. Indeed, the scope of these research techniques goes far beyond what we have described here, and our review should therefore be seen as a springboard for future research. For instance, these techniques can be used to examine other tobacco product marketing practices including point-of-sale displays, tobacco advertisements, and mass media campaigns. Similarly, neuroscience techniques can be applied to evaluate novel strategies for restricting, modifying, or banning tobacco advertisements or to study the effects of modified tobacco products and determine their likely impact. Beyond tobacco control, these techniques can answer policy-relevant research questions in other fields, such as the potential impacts of alcohol advertisements^78^ and food labeling.^79^

Rather than replacing more traditional methods of examining tobacco control measures, such as observational experiments, surveys, and questionnaires, neuroscience techniques should be a useful adjunct to these methods. These techniques can provide more objective evidence of the impacts of policy measures, allow investigation where it is not possible to conduct behavioral manipulations, and facilitate a deeper understanding of the cognitive mechanisms underlying the impacts of tobacco control policies. In addition, it seems that results from neuroscience studies can be more persuasive legally than those relying on more subjective methodologies. Although our research questions should not be solely led by their potential to have “impact,” this is an important consideration when working in a field with such clear implications for policy. We hope our review demonstrates that we can use the techniques developed in the consumer neuroscience literature that focus on the goal of *increasing* purchasing behavior and turn these on their heads to *reduce* tobacco purchasing.

## Funding

This work was supported in part by the Medical Research Council Integrative Epidemiology Unit at the University of Bristol, which is supported by the Medical Research Council and the University of Bristol (MC_UU_12013/6 and MC_UU_12013/7) and the Economic and Social Research Council (ES/R003424/1). JO was supported by the National Institute on Drug Abuse (K23 DA042898). The funders had no role in study design, data collection, and analysis, decision to publish, or preparation of the manuscript.

## Declaration of Interests

None declared.
